# Supplementary material for: Identification of photoperiod-induced specific miRNAs in the adrenal glands of Sunite sheep (Ovis aries)
Source: Front Vet Sci. 2022 Jul 22;9:888207. doi: 10.3389/fvets.2022.888207 (PMC9354845; doi:10.3389/fvets.2022.888207)
Supplement: Supplementary file 1 [file Data_Sheet_1.ZIP › Supplementary file/miRDeep2 file.zip!/miRDeep2 file/Know_Structure/SPLP42A1/oar-mir-27a.pdf]

miRBase precursor : oar-mir-27a
Total read count : 241538
oar-miR-27a read count : 241395
remaining reads : 143

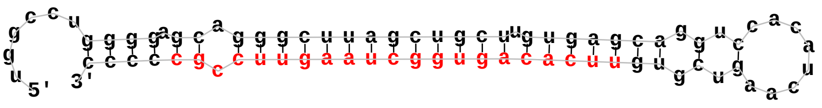

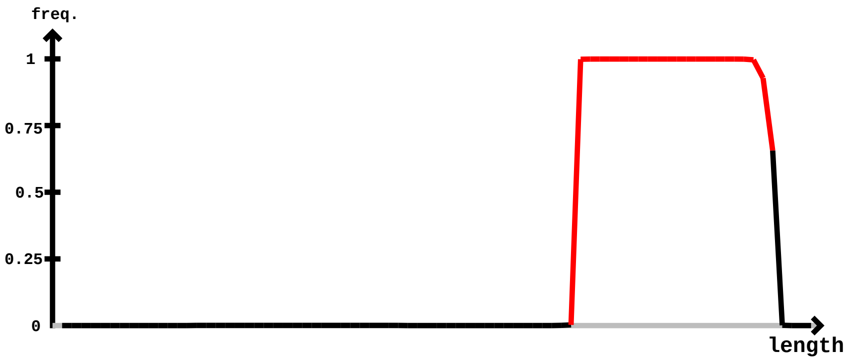

oar-miR-27a

```
5'- uggccuggggagcagggcuuagcugcuugugagcagguccacaucaagucguguucacaguggcuaaguuccgccccc  -3'    exp
    ......((((.((.(((((((((((((.(((((((.(.(........).).))))))))))))))))))))).))))))   reads      mm         sample
    ............agggcuuagcugcuugug................................................   1          0          seq
    ............agggcuuagcugcuugugag..............................................   1          0          seq
    ............agggcuuagcugcuugugagc.............................................   19         0          seq
    ............agggcuuagcugcuugugagca............................................   110        0          seq
    ............agggcuuagcugcuugugagcag...........................................   1          0          seq
    ............agggcuuagcugcuugugagcagg..........................................   7          0          seq
    ............agggcuuagcugcuugugagcaggu.........................................   2          0          seq
    ............agggcuuagcugcuugugagcagguc........................................   1          0          seq
    .............gggcuuagcugcuugugagcagg..........................................   1          0          seq
    ...................................................uguucacaguggcuaaguu........   1          0          seq
    ...................................................uguucacaguggcuaaguuc.......   6          0          seq
    ...................................................uguucacaguggcuaaguucU......   9          1          seq
    ...................................................uguucacaguggcuaaguucc......   13         0          seq
    ...................................................uguucacaguggcuaaguucUg.....   1          1          seq
    ...................................................uguucacaguggcuaaguuccg.....   144        0          seq
    ...................................................uguucacaguggcuaaguuccgc....   13         0          seq
    ...................................................uguucacaguggcuaaguuccgcc...   1          0          seq
    ....................................................guucacaguggcuaaguucc......   4          0          seq
    ....................................................guucacaguggcuaaguucU......   34         1          seq
    ....................................................guucacaguggcuaaguuccg.....   1          0          seq
    ....................................................guucacaguggcuaaguucUg.....   76         1          seq
    ....................................................guucacaguggcuaaguucUgc....   324        1          seq
    ....................................................guucacaguggcuaaguuccgcc...   1          0          seq
    .....................................................uucacaguggcuaaguG........   5          1          seq
    .....................................................uCcacaguggcuaaguu........   1          1          seq
    .....................................................uucacaAuggcuaaguu........   1          1          seq
    .....................................................uucacaguAgcuaaguu........   1          1          seq
    .....................................................uucacaguggcuaaguu........   70         0          seq
    .....................................................uucacaguggcuaagCu........   3          1          seq
    .....................................................uucacaguggcuaaguuG.......   6          1          seq
    .....................................................uucacaguggcuaaguuc.......   637        0          seq
    .....................................................uucacaguggcuaaguucU......   13795      1          seq
    .....................................................uucacaguggcuaaguucc......   2677       0          seq
    .....................................................uucacaguggcuaaguucUg.....   24429      1          seq
    .....................................................uucacaguggcuaaguuccg.....   33275      0          seq
```

oar-miR-27a

```
uggccuggggagcagggcuuagcugcuugugagcagguccacaucaagucguguucacaguggcuaaguuccgccccc
.....................................................uucacaguggcuaaguuccA.....   7681     1     seq
.....................................................uucacaguggcuaaguucUgc....   153475   1     seq
.....................................................uucacaguggcuaaguuccgc....   4240     0     seq
.....................................................uucacaguggcuaaguuccgcc...   266      0     seq
......................................................ucacaguggcuaaguucc......   2        0     seq
......................................................ucacaguggcuaaguucU......   12       1     seq
......................................................ucacaguggcuaaguucUg.....   17       1     seq
......................................................ucacaguggcuaaguuccA.....   3        1     seq
......................................................ucacaguggcuaaguuccg.....   19       0     seq
......................................................ucacaguggcuaaguuccgc....   5        0     seq
......................................................ucacaguggcuaaguucUgc....   117      1     seq
.......................................................cacaguggcuaaguucc......   1        0     seq
.......................................................cacaguggcuaaguucU......   2        1     seq
.......................................................cacaguggcuaaguuccA.....   1        1     seq
.......................................................cacaguggcuaaguucUg.....   1        1     seq
.......................................................cacaguggcuaaguuccg.....   2        0     seq
.......................................................cacaguggcuaaguucUgc....   16       1     seq
.......................................................cacaguggcuaaguuccgcc...   1        0     seq
........................................................acaguggcuaaguucUgc....   5        1     seq
.........................................................caguggcuaaguucUgc....   1        1     seq
```
